# A Multiple Rényi Entropy Based Intrusion Detection System for Connected Vehicles

**DOI:** 10.3390/e22020186

**Published:** 2020-02-06

**Authors:** Ki-Soon Yu, Sung-Hyun Kim, Dae-Woon Lim, Young-Sik Kim

**Affiliations:** 1 Major in Information Communication Engineering, Dongguk University, Seoul 04620, Koreadaewoonlim@gmail.com (D.-W.L.); 2 School of Computing, Korea Advanced Institute of Science and Technology, Daejeon 34141, Korea; harryzzong@kaist.ac.kr; 3 Department of Information and Communication Engineering, Chosun University, Gwangju 61452, Korea

**Keywords:** connected vehicles, intrusion detection system (IDS), Rényi entropy, Shannon entropy, vehicular network

## Abstract

In this paper, we propose an intrusion detection system based on the estimation of the Rényi entropy with multiple orders. The Rényi entropy is a generalized notion of entropy that includes the Shannon entropy and the min-entropy as special cases. In 2018, Kim proposed an efficient estimation method for the Rényi entropy with an arbitrary real order α. In this work, we utilize this method to construct a multiple order, Rényi entropy based intrusion detection system (IDS) for vehicular systems with various network connections. The proposed method estimates the Rényi entropies simultaneously with three distinct orders, two, three, and four, based on the controller area network (CAN)-IDs of consecutively generated frames. The collected frames are split into blocks with a fixed number of frames, and the entropies are evaluated based on these blocks. For a more accurate estimation against each type of attack, we also propose a retrospective sliding window method for decision of attacks based on the estimated entropies. For fair comparison, we utilized the CAN-ID attack data set generated by a research team from Korea University. Our results show that the proposed method can show the false negative and positive errors of less than 1% simultaneously.

## 1. Introduction

In modern cars, dozens of electronic control units (ECUs) are operated together, and they communicate over controller area networks (CANs). The connectivity between cars and the Internet will be further accelerated by the advancement of smart and autonomous vehicles. Increasing connectivity can help improve performance or convenience; however, modern vehicles have become more vulnerable to hacking attacks owing to this. Automotive systems based on CAN bus are already in common use, but no security considerations against hacking have been made since the design of the protocol. Since 2010, many instances of car hacking through the on-board diagnostics II (OBD-II) port for in-vehicle diagnosis and the infotainment system have been reported [1,2,3,4,5,6,7,8]. Thus, security measures for modern cars against hacking threats also have been actively researched [7,9,10]. Because vehicular security is an important issue today, we need a reliable solution to protect vehicles in motion. Here, we also need a lightweight algorithm since the available devices have very constrained computational power. In this paper, we propose a solution for a vehicular intrusion detection system (IDS) with both low complexity and reliability simultaneously.

### Related Works

Network IDS (NIDS) is an important piece of network security equipment. Recently, machine learning based IDSs for general networks have been proposed for malware classification [11,12] and intrusion detection [13], which are based on a deep belief neural (DBN) network and can detect unknown attacks up to 97.5% of the time. However, a large dataset is necessary for the training process. For intrusion detection, entropy is also an important measure for anomaly. In [14], several kinds of entropy definitions, such as Shannon entropy, Rényi entropy, and Tsallis entropy, were used to detect intrusion at the same time. Then, it was improved by combining wavelet and principal component analysis with previous entropy measures [15]. In this paper, we focus on Rényi entropy in order to maintain low computational complexity in the vehicular environment. Additionally, a discretized extended feature space (DEFS) model is used for IDS [16], wherein the number of event patterns can be reduced by grouping similar patterns based on feature values. There is a probabilistic-driven ensemble (PDE) approach that operates by using several classification algorithms, whose effectiveness has been improved on the basis of a probabilistic criterion [17]. A series of experiments, performed by using real-world data, show how such an approach outperforms the state-of-the-art competitors, proving its better capability to detect intrusion events with regard to the canonical solutions.

To secure vehicular environments, two different approaches have been considered: security countermeasures based on encryption and authentication for data over vehicular networks, and intrusion detection systems that can detect suspicious activities on the networks [18,19,20,21,22,23,24,25,26,27]. In this work, we focus on the intrusion detection system based on the estimation of the Rényi entropy with multiple orders [28]. The Rényi entropy is a generalized notion of entropy that includes the Shannon entropy and the min-entropy as special cases [29].

In 2018, Kim proposed an efficient estimation method for Rényi entropy with arbitrary real order α [28]. In this work, we utilize this method to construct a multiple order Rényi entropy based intrusion detection system (IDS) for vehicular systems with various network connections. The proposed method estimates the Rényi entropies with three distinct orders, two, three, and four, simultaneously, based on the CAN-IDs of consecutively generated frames. The collected frames are split into blocks with fixed numbers of frames, and the entropies are evaluated based on these blocks. For a more accurate estimation against each type of attack, we also propose a retrospective sliding window (RSW) method on the estimated entropy values. For fair comparison, we utilized the CAN-ID attack data set generated by a research team from Korea University [30]. Our results show that the proposed method can show the false negative and positive errors of less than 1% simultaneously.

The rest of the paper is organized as follows. In Section 2, the basic definitions and notion are defined for the understanding of the proposed scheme. Additionally, two main attack models considered in this paper are explained. In Section 3, the theoretical analysis of Rényi entropy with respect to attack rate is presented. In addition, the proposed algorithm to measure multiple order Rényi entropies simultaneously and the improvements based on RSW method are provided. In Section 4, simulation results based on the vehicular attack data set are discussed. Finally, we conclude this paper in Section 5.

## 2. Preliminaries

The basic principle of an IDS for a vehicular system is the same as that of an IDS for a general network [31]. The first method requires storing pre-specified signatures of external attacks, inspection of transmitted packets, and analyzing whether any pattern matches with the stored signatures. The second method detects abnormalities using statistical characteristics of the normal range of the data generated by the vehicle.

One of the biggest differences between conventional networks and vehicular networks in the viewpoint of IDS is that messages generated and transmitted in intra-vehicular networks have uniform and regular characteristics, because the traffic usually conveys control or status information of the machine, unlike those made by humans over general networks. Because estimation is made by determining whether the abnormal phenomenon is normally deviated from the pattern, the probability of error can be reduced, compared to general networks. Meanwhile, the computational power of the ECUs used in vehicles is limited compared to general network environments, and thus, complicated algorithms are not adequate in ECU environments. The time required to collect enough packets should also be minimized.

Entropy-based IDSs have been proposed, because the entropy measure can reflect the statistical characteristics of the traffic over networks [10,18]. Entropy based detection methods for intrusions have already been applied to IDSs for general networks, and these possibilities have been considered for vehicular environments. For example, [10] proposed an intrusion detection method using relative distance (RD) and conditional self-information (CSI) for vehicular networks. RD is the probability distribution of two sets of events, and is defined as

$$\begin{array}{c} RD_{p|q}\left(x\right)=p\left(x\right)\log_{2}\frac{p(x)}{q(x)}. \end{array}$$

As the name suggests, RD can be used as a metric to determine the relative distance between two probability distributions. If q(x) denotes the distribution of the normal intra-vehicular network traffic and p(x) denotes the distribution of the current intra-vehicular network traffic, then the large value of $RD_{p|q}\left(x\right)$ gives the distance between p(x) and q(x). That is, it indicates that the current traffic represented by p(x) is far from the normal one.

Entropy can be used as an indicator of abnormality in internal data of vehicles. However, to estimate the entropy of data sets generated in real time, the entropy can be calculated only after collecting enough data sets. This is because the distributions p(x) and q(x) should be available before $RD_{p|q}\left(x\right)$ can be calculated. It may not respond immediately to real time attacks since it has a complicated operation to deal with cumulative value of logarithms.

### 2.1. Shannon Entropy and Rényi Entropy

The first and the most popular definition of entropy for information is Shannon entropy [32]. Let $F_{2}$ be the finite field with two elements {0,1}. Now, suppose that *X* is an *L*-bit output random variable from a random source S. Then, the Shannon entropy is defined as

$$\begin{array}{c} H\left(X\right)=-\sum_{b\in F_{2^{L}}}\mathrm{Pr}\left(b\right)\log_{2}\mathrm{Pr}\left(b\right). \end{array}$$

In this work, the random source S corresponds to a CAN system, and *X* corresponds to the continuously generated CAN-IDs over the network. *L* is given by $\left\lceil \log_{2}M\right\rceil$, where *M* is the total number of used CAN-IDs. Vehicular IDSs based on Shannon entropy have been proposed.

In 1961, a more generalized definition of entropy for information was proposed by Rényi [29]. Because this generalized definition includes the Shannon entropy and the min-entropy (another popular entropy measure) as special cases, it has been utilized in many applications. The Rényi entropy is defined as

$$\begin{array}{c} R_{\alpha }=\frac{1}{1-\alpha }\log_{2}\sum_{b\in F_{2^{L}}}\mathrm{Pr}{\left(b\right)}^{\alpha } \end{array}$$

where α>0 and α≠1.

### 2.2. Efficient Estimation of Rényi Entropy

Recently, for real values of the order (α>0 and α≠1), Kim proposed an efficient estimation of Rényi entropy, based on the distance to the nearest neighbor [28]. For the estimation, he defined a test function $f(s^{N})$ for a random sample $s^{N}$ of length *N* as

$$\begin{array}{c} f\left(s^{N}\right)=\frac{1}{K}\sum_{n=1}^{K}g\left(D_{n}\left(s^{N}\right)\right) \end{array}$$

where $D_{n}\left(s^{N}\right)$ is the minimum distance between the current sample and the previous sample with the same CAN-ID as the current one. For the estimation of Rényi entropy of order α, the parameter g(k) of the estimator for a given index distance *k* is given by

$$\begin{array}{c} g\left(k\right)=\left\{\begin{array}{cc} 1, & \mathrm{if}\;k=1\\ {\left(-1\right)}^{k-1}P_{k-1}^{\alpha -2}, & \mathrm{if}\;k\geq 2 \end{array}\right. \end{array}$$

where

Pk−1α−1=α−2k−1=(α−2)(α−3)⋯(α−k)(k−1)!.

For α=2, the parameter g(x) is given by

$$\begin{array}{c} g\left(x\right)=\left\{\begin{array}{cc} 1, & \mathrm{if}\;k=1\\ 0, & \mathrm{otherwise}. \end{array}\right. \end{array}$$

For α=3, the parameter g(x) is given by

$$\begin{array}{c} g\left(x\right)=\left\{\begin{array}{cc} 1, & \mathrm{if}\;k=1\\ -1, & \mathrm{if}\;k=2\\ 0, & \mathrm{otherwise}. \end{array}\right. \end{array}$$

Finally, for α=4, the parameter g(x) is presented as

$$\begin{array}{c} g\left(x\right)=\left\{\begin{array}{cc} 1, & \mathrm{if}\;k=1\\ -2, & \mathrm{if}\;k=2\\ 1, & \mathrm{if}\;k=3\\ 0, & \mathrm{otherwise}. \end{array}\right. \end{array}$$

### 2.3. Attack Models

Originally developed by Bosch in 1983, CAN is a protocol designed for communication between micro-controllers. It was put in vehicles for the first time in 1989. CAN minimizes the cost of intra-vehicular communication, and it was standardized in 1993 by ISO as an international standard (ISO 11898). In vehicular applications, CAN has been used for connections and communications between engine management systems, transmission control systems, on-board controllers, and miscellaneous ECUs. It is possible to connect 2031 devices in a single network simultaneously. The CAN frame size varies from 44 bits to 108 bits, depending on the length of payload. Because the maximum bandwidth of CAN is 1 Mbps, it is possible to transmit 9259 frames in a second when the frame size is fixed as 108 bits.

An open CAN attack data set created by Korea University in 2017 [30] is utilized to evaluate the proposed IDS in the CAN environment. In this data set, two major attack types are considered, the denial of service (DoS) attack and the fuzzy attack. In accordance with CAN specifications, the CAN-IDs with lower values have higher priorities. Therefore, for the DoS attack, high priority CAN-IDs are intentionally injected into the network to prevent transmission of the normal network traffic. In case of the fuzzy attack, randomly generated CAN-IDs are continuously injected along with the normal traffic to interrupt the normal data transmission.

## 3. Theoretical Analysis of Entropy with Respect to Attack Rate

In this section, we analyze the values of the Rényi entropies theoretically against two attack models, the DoS attack and the fuzzy attack.

### 3.1. Case 1: DoS Attack

In case of the DoS attack, we assume that the CAN-ID with zero (the highest priority CAN-ID) is used in the attack frame. If the CAN-ID with zero is continuously injected, the other frame does not have any chance to transmit its data, owing to the priority. We first assume that there are *K* distinct CAN-IDs with distributions $\left(n_{0},n_{1},\ldots ,n_{K}\right)$, each corresponding to CAN-ID 0 to *K*, in order.

If there is no attack (normal phase), we have $n_{0}=0$. Therefore, the total number of frames injected into the network is $N=\sum_{i=0}^{K}n_{i}$. Let $\left(d_{0},d_{1},\ldots ,d_{K}\right)$ be the differences between the frequencies of each CAN-ID in the normal phase and in the attack phase. In this setting, if we add all of the differences, we have $\sum_{i=0}^{K}d_{i}=0$. Then, we can represent the Rényi entropy $E_{A}\left(\beta \right)$ in the attack phase using the Rényi entropy in the normal phase *E*, where the attack rate β is given by $\beta =n_{0}/N$.

**Theorem** **1.** *In the DoS attack scenario, the Rényi entropy with order α is defined as*

(1) $$\begin{array}{c} E\left(\beta \right)=-\frac{1}{1-\alpha }\log_{2}\left({\left(1-\beta \right)}^{\alpha }2^{-(1-\alpha )E}+\beta^{\alpha }\right). \end{array}$$

**Proof.**  In the DoS attack model, the frequency of the attack CAN-ID with the highest priority increases, and the frequencies of the other CAN-IDs decrease. Thus, we have the following relation:

$$\begin{array}{c} d_{0}=-\sum_{i=1}^{K}d_{i}\;\mathrm{where}\;d_{0}>0\;\mathrm{and}\;d_{i}<0\;\mathrm{for}\;1\leq i\leq K. \end{array}$$

Then, the relative rates of the CAN-IDs (except for the attack ID) decrease with the attack rate β. Therefore, we have $d_{i}=-\beta n_{i}$, for 1≤i≤K. Thus, the probability of occurrence for each CAN-ID follows the distribution $\left(p_{0},\ldots ,p_{K}\right)=\left\{\frac{n_{0}}{N},\frac{n_{1}}{N},\ldots ,\frac{n_{K}}{N}\right\}$. Thus, the Rényi entropy with order α for the normal phase is given by

(2) $$\begin{array}{c} E=\frac{1}{1-\alpha }\log_{2}\left(\frac{\sum_{i=0}^{K}n_{i}^{\alpha }}{N^{2}}\right). \end{array}$$

Similarly, the Rényi entropy with order α for the attack phase is defined as

(3) $$\begin{array}{c} E_{A}\left(\beta \right)=\frac{1}{1-\alpha }\log_{2}\left(\frac{\sum_{i=0}^{K}{\left(n_{i}+d_{i}\right)}^{\alpha }}{N^{2}}\right). \end{array}$$

Then,

(4) $$\begin{array}{cc} \sum_{i=0}^{K}{\left(n_{i}+d_{i}\right)}^{\alpha } & =\sum_{i=1}^{K}{\left(n_{i}-\beta n_{i}\right)}^{\alpha }+{\left(\beta N\right)}^{\alpha } \end{array}$$

(5) $$\begin{array}{cc} \; & ={\left(1-\beta \right)}^{\alpha }\sum_{i=1}^{K}{n_{i}}^{\alpha }+N^{\alpha }\beta^{\alpha }. \end{array}$$

Therefore, from the definition of Rényi entropy in (2), we have

$$\begin{array}{cc} \frac{\sum_{i=0}^{K}{\left(n_{i}+d_{i}\right)}^{\alpha }}{N^{\alpha }} & ={\left(1-\beta \right)}^{\alpha }\frac{\sum_{i=1}^{K}n_{i}^{\alpha }}{N^{\alpha }}+\beta^{\alpha }\\ \; & ={\left(1-\beta \right)}^{\alpha }2^{-(1-\alpha )E}+\beta^{\alpha }. \end{array}$$

Finally, we have

$$\begin{array}{c} E\left(\beta \right)=\frac{1}{1-\alpha }\log_{2}\left({\left(1-\beta \right)}^{\alpha }2^{(1-\alpha )E}+\beta^{\alpha }\right). \end{array}$$

 □

### 3.2. Case 2: Fuzzy Attack

We now assume that the CAN-IDs are generated randomly, according to the uniform distribution. Suppose that the total number of occupied normal CAN-IDs is *k*. Owing to the randomly generated attack CAN-IDs, the total number of used CAN-IDs increases up to *K*, where K≥k.

In the normal phase, the distribution of the CAN-IDs is given by $n_{1},n_{2},\ldots ,n_{k},n_{k+1},\ldots ,n_{K}$, where $n_{k+1}=\ldots =n_{K}=0$. Then, we have $N=\sum_{i=1}^{k}n_{i}$, which corresponds to the total number of frames. Let the numbers of occurrences of individual CAN-IDs in the attack be $\left(d_{1},\ldots ,d_{K}\right)$, and let β be the attack rate defined by $\beta =\sum_{i=1}^{K}d_{i}/N$.

Because the CAN IDs injected by the attacker are uniformly and randomly generated, we can assume that $n_{i}=\frac{\beta N}{K}$ for 1≤i≤K. Because the attack can interrupt and collide with the normal transmission, we assume the reduced frequency of the normal CAN-IDs due to the attack is given by $c_{1},c_{2},\ldots ,c_{K}$, such that $c_{k+1}=c_{k+2}=\ldots =c_{K}=0$. If we assume that each CAN-ID has similar reduced rates due to the uniform random injection of each CAN-ID, we have $c_{i}=\beta N_{i}$. Again, for the Rényi entropy of order α for the normal phases, we have

$$\begin{array}{c} E_{\alpha }=\frac{1}{1-\alpha }\log_{2}\frac{\sum_{i=1}^{K}n_{i}^{\alpha }}{N^{\alpha }}=\frac{1}{1-\alpha }\log_{2}\left(\frac{\sum_{i=1}^{k}n_{i}^{\alpha }}{N^{\alpha }}\right). \end{array}$$

**Theorem** **2.** *For given Rényi entropies $E_{2}$, $E_{3}$, and $E_{4}$ of orders two, three, and four, respectively, in the normal phase, we have the corresponding Rényi entropies in the attack phase, $E_{2}\left(\beta \right)$, $E_{3}\left(\beta \right)$, and $E_{4}\left(\beta \right)$ of orders two, three, and four, with attack rate β as follows:*

*(1) For α=2, we have*

$$\begin{array}{c} E_{2}\left(\beta \right)=-\log_{2}\left({\left(1-\beta \right)}^{2}2^{-E_{2}}+\frac{\beta (2-\beta )}{K}\right); \end{array}$$

*(2) For α=3, we have*

$$\begin{array}{c} E_{3}\left(\beta \right)=-\frac{1}{2}\log_{2}\left({\left(1-\beta \right)}^{3}2^{-2E_{3}}+3\frac{\beta {\left(1-\beta \right)}^{2}}{K}2^{-E_{2}}+\frac{\beta^{2}}{K^{2}}\left(3-2\beta \right)\right); \end{array}$$

*(3) For α=4, we have*

$$\begin{array}{c} E_{4}\left(\beta \right)=-\frac{1}{3}\log_{2}\left({\left(1-\beta \right)}^{4}2^{-3E_{4}}+4\frac{\beta {\left(1-\beta \right)}^{3}}{K}2^{-2E_{3}}+6\frac{\beta^{2}{\left(1-\beta \right)}^{2}}{K^{2}}2^{-E_{2}}+\frac{\beta^{3}}{K^{3}}\left(4-3\beta \right)\right). \end{array}$$

**Proof.**  For the attack phase, we can represent the Rényi entropy as follows:

$$\begin{array}{c} E_{\alpha }\left(\beta \right)=-\frac{1}{1-\alpha }\log_{2}\left(\frac{\sum_{i=1}^{K}{\left(n_{i}+d_{i}-c_{i}\right)}^{\alpha }}{N^{\alpha }}\right). \end{array}$$

The inner summation of the logarithm is given by

(6) ∑i=1K(ni+di−ci)α=∑i=1k(ni+βNK−βni)α+(K−k)βNKα=∑i=1k((1−β)ni+βNK)α+(K−k)βNKα=∑i=1k∑j=0ααj((1−β)ni)jβNKα−j+(K−k)βNKα=βNKα∑j=0ααj(1−β)jβNK−j∑i=1knij+(K−k)βNKα=βNKα∑j=0ααj1−ββjKNj∑i=1knij+(K−k).

By applying α=2,3,4 to (6), the statements of this theorem are proven. □

### 3.3. Proposed Algorithm for Estimation of Rényi Entropies with Orders 2, 3, and 4

The proposed IDS scheme identifies intrusions based on the estimated Rényi entropies and the changing patterns of the estimated entropy values for block-wise data. The continuously produced CAN frames are split into blocks, and the estimated values are updated on the outstanding blocks via the RSW method to quickly produce intermediate entropy values. Based on the estimation method for the Rényi entropies described in Section 2.2 [28], we can formulate Algorithm 1 for estimating Rényi entropies with three distinct orders 2, 3, and 4 simultaneously, using the blocks with fixed lengths $N_{S}$, which contain consecutive $N_{S}$ frames over the CAN.
**Algorithm 1:** Proposed estimation of Rényi entropy with multiple orders 2, 3, and 4.
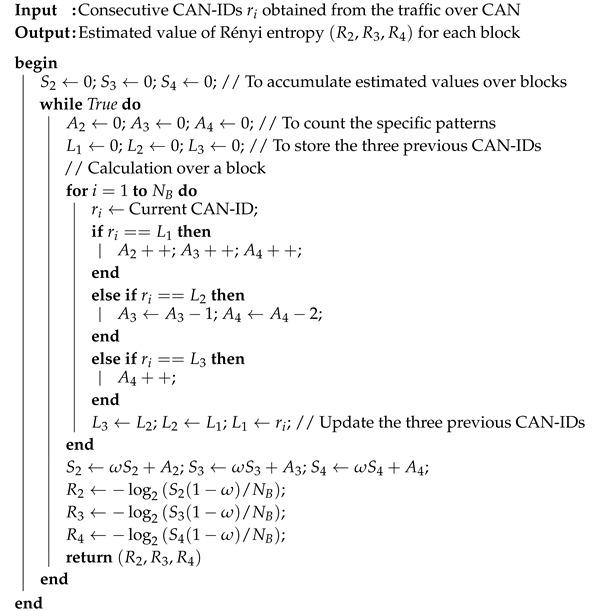


### 3.4. Improving Accuracy Using a Characterizing Attack Pattern with RSW

In the proposed scheme, attacks can be detected using the Rényi entropies derived from the frequencies of individual CAN-IDs embedded in the CAN frames. In this subsection, we explain the parameters and criteria for the detection, such as block size, acceptable range, and RSW size. Usually, it is not possible to detect intrusions immediately because of the estimation of Rényi entropy. To overcome this shortcoming, we propose a retrospective way of decision-making that can improve the missing probability of the proposed IDS. This will be used to audit and analyze the attack features after the attack or central monitoring server to prevent similar attacks later.

**Figure 1:**
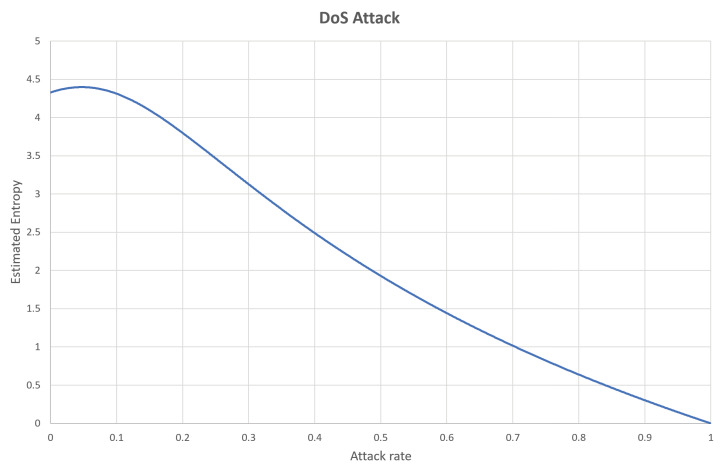
Entropy changes with respect to the attack rate of the denial of service (DoS) attack.

To enhance the accuracy of detection, we can also utilize the characteristics of specific attacks, such as the DoS attack and fuzzy attack. The DoS attack exploits the order of priorities of the CAN frames. Therefore, if the highest order CAN-ID is consecutively inserted into the network, the normal traffic does not have any chance to transmit. However, in this case, the estimated entropy is significantly reduced owing to the reduction in randomness (uncertainty). This can be observed in the sample test data obtained from Korea University, as shown in Figure 1 [30].

In Figure 1, the x-axis represents the attack ratio (the attack frames with respect to the normal traffic) and the y-axis represents the estimated entropy value. For the normal traffic, the estimated entropy value is around 4.3. However, when the attack is applied, the estimated value increases slightly and is then reduced significantly with the attack rate. If the attack rate is around 5–10% (that is, if new CAN-IDs of 5–10% are inserted), the traffic becomes diverse compared to the normal traffic. This is because the newly inserted CAN-IDs are similar in number to the other CAN-IDs. However, the inserted frames become the majority and reduce the diversity and randomness of the CAN-IDs, because a significant number of CAN-IDs are of the highest priority. The estimated entropy is thus significantly reduced.

**Figure 2:**
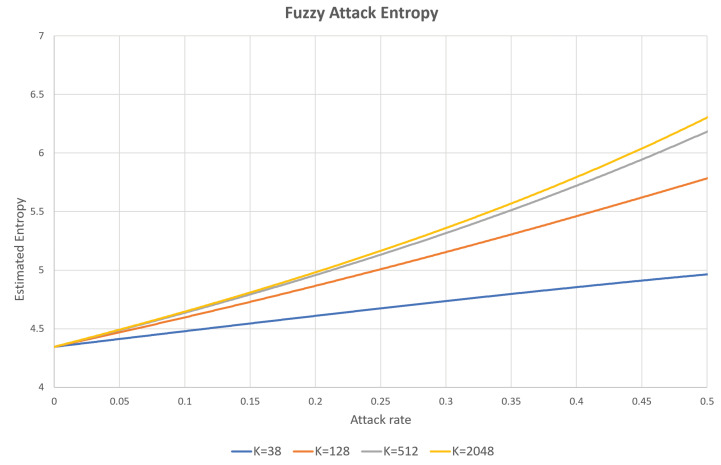
Entropy changes with respect to the attack rate of the DoS attack.

Fuzzy attack is another type of attack in which randomly generated CAN frames with random CAN-IDs are inserted into the network. This attack exploits the fact that any ECU will accept any CAN frame with an ID in the proper range. Therefore, the fuzzy attack will increase the estimated entropy, according to the increase in the attack rate. This phenomenon can be observed in Figure 2, which is based on the same test CAN traffic generated from the data set from Korea University [30]. As can be seen in the figure, the estimated values increase with the attack rate and the range of the CAN-IDs.

The block size defines the number of CAN frames used in the evaluation of the Rényi entropy, and the indices of CAN frames in a block move according to the RSW method. The Rényi entropy is evaluated using the frequencies of individual CAN-IDs in a block. If the evaluated Rényi entropy is not in the allowed range, it is implied that there is an intrusion.

To reduce false alarms and missing probabilities in DoS and fuzzy attacks, we propose a new method that utilizes the fluctuation of Rényi entropy values after the attack. These values can be used to audit and analyze the attack behavior in a central analysis center to develop a countermeasure to the attack, and to prevent similar future attacks.

Using the dependence of the entropy behavior on the class of attacks, we can reduce the missing and false alarm probabilities simultaneously. The proposed intrusion detection method is described in the following eight steps, which are carried out repeatedly.

Step 1. Generate an *i*-th block, $blk_{i}$, by accumulating CAN-IDs of the sequentially generated $N_{B}$ frames into the queue of CAN-IDs, $que_{id}$, where $N_{B}$ is the size of a block.
Step 2. Entropy $h_{i}$ related to the frequencies of the individual CAN-IDs accumulated in $que_{id}$ in Step 1 is evaluated.
Step 3. By comparing the estimated entropy $h_{i}$ in Step 2 with the pre-specified normal entropy *H*, the first decision of whether $blk_{i}$ is normal or abnormal is made. Denote $d_{i}=0$ for the normal block and $d_{i}=1$ otherwise.
Step 4. Find the entropy change information $p_{i}$, by comparing $h_{i}$ with $h_{i-1}$ according to the following rules:
  $$\begin{array}{c} p_{i}=\left\{\begin{array}{cc} 0, & \mathrm{if}\;h_{i}=h_{i-1}\\ \sigma_{0}, & \mathrm{if}\;h_{i}>h_{i-1}\;\mathrm{and}\;d_{i}=0\\ -\sigma_{0}, & \mathrm{if}\;h_{i}<h_{i-1}\;\mathrm{and}\;d_{i}=0\\ \sigma_{1}, & \mathrm{if}\;h_{i}>h_{i-1}\;\mathrm{and}\;d_{i}=1\\ -\sigma_{1}, & \mathrm{if}\;h_{i}<h_{i-1}\;\mathrm{and}\;d_{i}=1, \end{array}\right. \end{array}$$
  where $\sigma_{0}\leq \sigma_{1}$.
Step 5. $p_{i}$ is stored in the pattern queue $que_{patt}$.
Step 6. Check whether the pattern of entropy change information $p_{i}$s in $que_{patt}$ fits into the rules specified according to the types of attacks, and whether the pattern matches with one of the rules. These blocks are treated as attack patterns, even if the result of the first decision is classified as normal traffic.
Step 7. Otherwise, if there is no matching rule among the $P_{size}$ amount of consecutive $p_{i}$s, $P_{size}$ blocks are removed from $que_{pat}$. In this case, the intrusion decision on the output blocks is determined by the results of the first decision.
Step 8. CAN-IDs in the considered block slide (i.e., the number of blocks in a slide multiplied by $N_{S}$) are removed from $que_{id}$.

**Figure 3:**
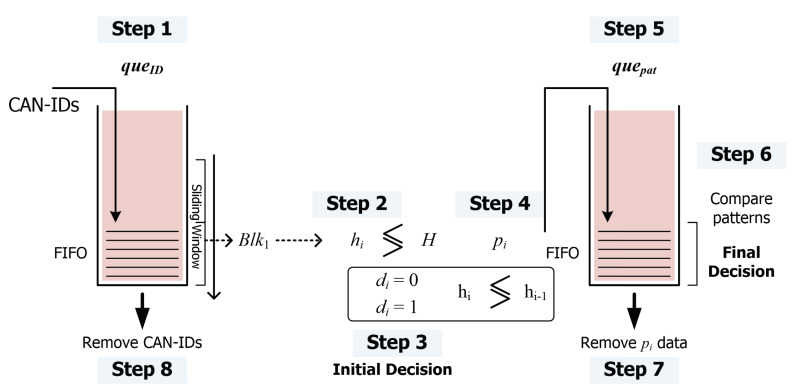
Proposed RSW method based on the estimated entropies.

This process is depicted in Figure 3. Therefore, we can define the entropy change to be a pattern for specific attacks. If pre-specified patterns are observed in the transmitted traffic, the miss rate can be reduced, because we can detect an attack even if the entropy is still in the allowed range.

**Figure 4:**
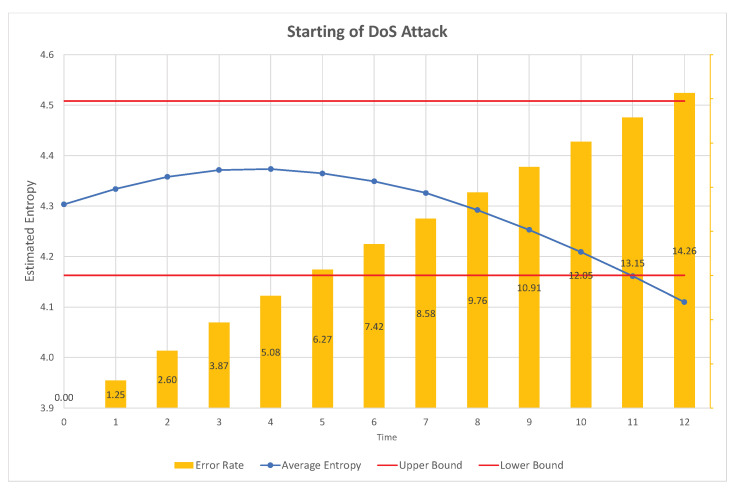
Error rate with respect to the size of the RSW.

**Figure 5:**
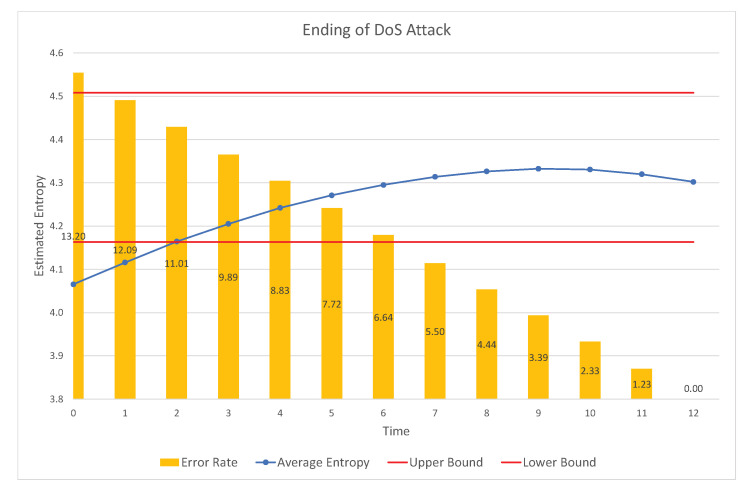
Error rate with respect to the size of the RSW.

Because the data over CAN is generated continuously during the operation of the vehicle, the data will be split into blocks with the fixed number of CAN frames. Then, the number of frequencies of individual CAN-IDs in a block will be counted to estimate the Rényi entropy of the generated CAN data. If the estimated Rényi entropy is not in the approved normal range, it will be treated as an abnormal block. In this work, we propose a more accurate method to detect intrusions by distinguishing suspicious blocks that are in the approved range, but may correspond to the initial frames of the attack. This will be determined based on the real-time behavior of the estimated Rényi entropy. Figure 4 and Figure 5 show that the examples of detected abnormal frames in the proposed IDS, based on real-time changes of the Rényi entropy.

## 4. Numerical Results

**Figure 6:**
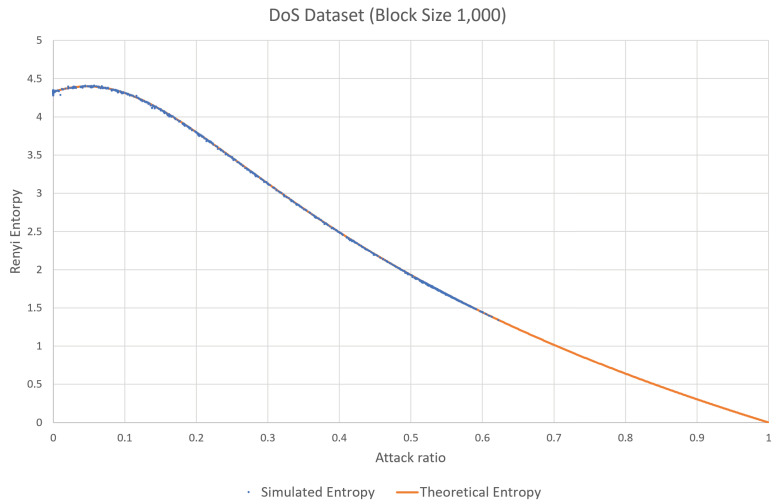
Comparison between the theoretical expectation and the result from the real data set in the DoS attack.

**Figure 7:**
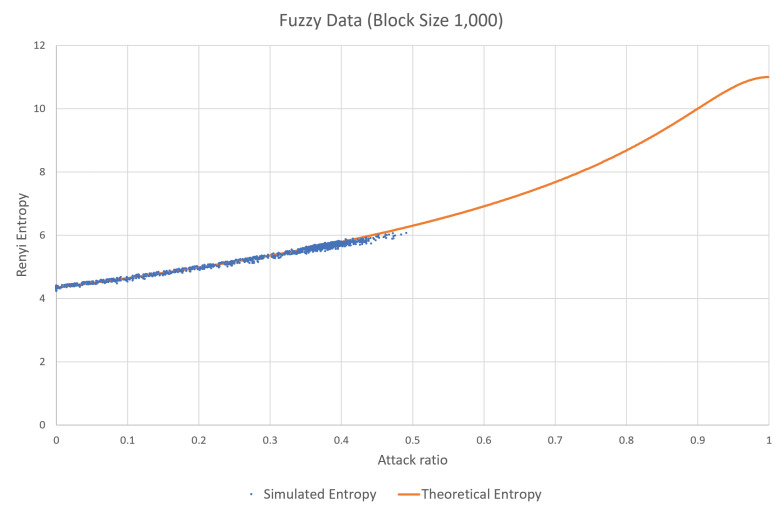
Comparison between the theoretical expectation and the result from the real data set in the fuzzy attack.

**Figure 8:**
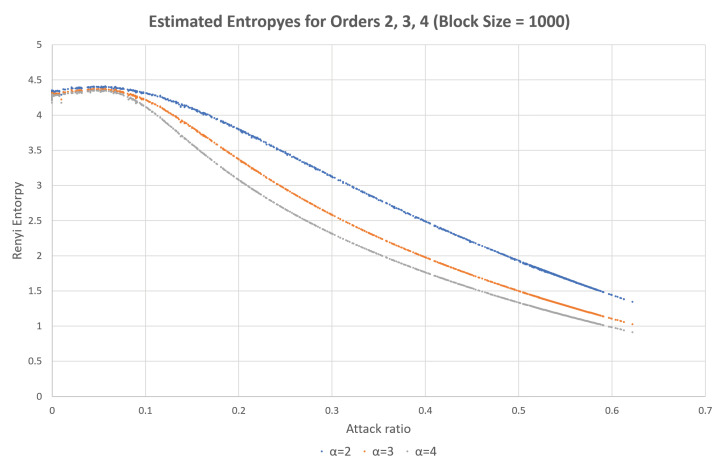
Estimated Rényi entropies with orders 2, 3, and 4 with respect to the attack rate for the DoS attack.

**Figure 9:**
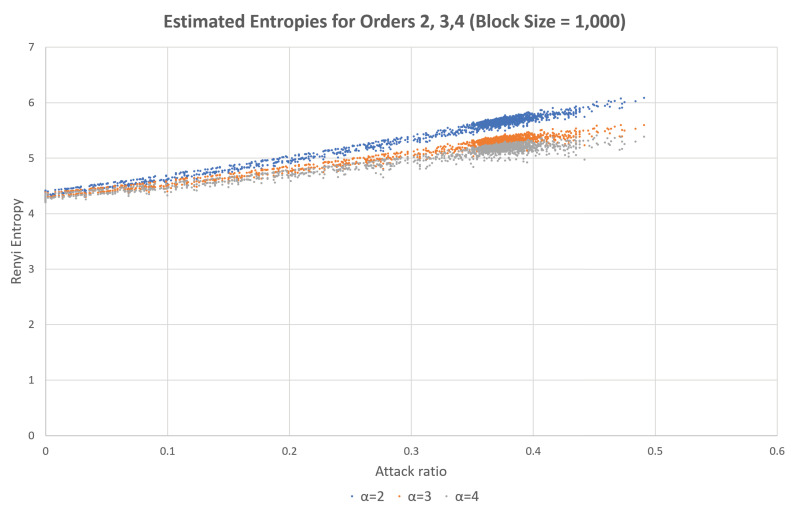
Estimated Rényi entropies with orders 2, 3, and 4 with respect to the attack rate for the Fuzzy attack.

In the dataset provided by Korea University [30], the interval without attack and the interval with attack existed together. After splitting data blocks, we calculated attack rate and estimated entropies for individual blocks. Then, the same attack rate values were collected and sorted, and the corresponding estimated entropy values are presented as in Figures 1, 2, 4, 5, 8 and 9. Contrary to this, Figure 6 and Figure 7 directly use the dataset.

First, the accuracy of the theoretical analysis in Section 3.1 and Section 3.2 is depicted in Figure 6 and Figure 7. In these figures, the theoretical expectation is represented as an orange solid line with respect to the attack rate from 0 to 1, and the estimated entropy from the real attack data set is presented as a collection of blue dots. In the real attack data set for the DoS attack, the attack rate is distributed from 0 to 0.6 since the attack rate of 0.5 means that the half of the bandwidth is already occupied by the attacker and the higher attack rate is difficult to see in the real vehicles. Similarly, in the attack data set for the fuzzy attack, the attack rate is distributed from 0 to 0.5. Resultantly, the theoretical expectation and the estimated data from the real attack data set are almost overlapping the entire available range in both attack models.

Next, the RSW method is analyzed in Figure 4 and Figure 5. In a DoS attack, the entropy decreases continuously during the elapsed time due to the injection of the same CAN-IDs; i.e., due to reduced randomness. However, at the starting point, the entropy is in the allowed range. Thus, we can determine some CAN-IDs in the allowed range as attack frames, by observing the patterns of the decrease. The problem is in determining how many frames are classified as attack frames. Figure 4 shows the error rate with respect to the depth of the trace back. In this experiment, the attack starts at time 0. Therefore, if we determine the attack or intrusion at time 12, we miss many attack frames injected during time 0 to 12. Based on the movement of curve of estimated values, we can decide on more frames in the allowed range (i.e., time 0 to 12 in this example), so we can reduce the miss rate. For example, at the unit time 12, if we can decide that frames at the time 1 are also classified as attack frames, then the error rate (miss rate) is only 1.25 %. Contrary to this, we can also set the ending point more accurately, as in Figure 5. In this case, we can decide the frames in the time range 0 to 12 are attack frames.

In Figure 8 and Figure 9, the multiple estimated values are depicted at the same time by using the same dataset provided by Korea University [30]. We can set the different bounds for each estimated values and then decides whether there is an intrusion or not by observing estimated values. Among three estimated values, if more then two values are in the unallowed ranges and one value is still in the allowed range, then we can decide that there is an intrusion by using the majority vote rule.

Table 1 compares the false alarm rates and miss rates when we only use the estimated entropy, and utilize both estimated entropy and the entropy change patterns, respectively. It can be seen that the miss rate can be reduced by up to 54.6% and 32.6%, with similar false-alarm rate in DoS attack and fuzzy attack, respectively. This method can influence to increasing false alarm rate slightly because it designates previous frames as attack frames depending on the change patterns of estimated entropies.

## 5. Conclusions

In this work, we tested an IDS for vehicular networks by using multiple order Rényi entropies simultaneously. The proposed IDS considers several orders of Rényi entropy simultaneously. Each Rényi entropy can be estimated simultaneously with very low complexity. Estimated data can be used to detect anomalies in the intra-vehicular network traffics generated by the vehicle. During the estimation the collected frames were split into blocks with fixed number of frames, and the entropies were evaluated based on these blocks. For a more accurate estimation against each type of attack, we also propose a RSW method for decision of attacks based on the estimated entropies. For fair comparison, we utilized the CAN-ID attack data set generated by a research team from Korea University [30]. Our results show that the proposed method can show the false negative and positive errors of less than 1% simultaneously.

As further work, we will study IDS based on machine learning (ML) and improve the performance by applying estimated Rényi entropy to several ML algorithms. In addition, the proposed method was only simulated and validated on the two major attack models in the dataset provided by Korea University. However, it is necessary to verify the validity of the proposed scheme for other attack models, which will be possible after obtaining a valid dataset from an actual vehicular environment. This will be done in the future. Since the proposed method has very low complexity, it can be used for vehicle IDS and contributes to vehicle safety by enabling rapid detection of external attacks.

## Figures and Tables

**Table 1:** False alarm rates and the miss rate for the entropy case and entropy + RSW case with respect to the DoS attack and fuzzy attack.

**DoS Attack**	**False Alarm**	**Missing**
Entropy	0.78 (100) %	1.08 (100) %
Entropy + RSW	0.79 (101.3)%	0.49 (154.6)%
**Fuzzy Attack**	**False Alarm**	**Missing**
Entropy	0.39 (100) %	0.86 (100)%
Entropy + RSW	0.41 (105.1)%	0.58 (132.6)%
